# Oil Palm and Rubber Tree Water Use Patterns: Effects of Topography and Flooding

**DOI:** 10.3389/fpls.2017.00452

**Published:** 2017-04-03

**Authors:** Afik Hardanto, Alexander Röll, Furong Niu, Ana Meijide, Hendrayanto, Dirk Hölscher

**Affiliations:** ^1^Tropical Silviculture and Forest Ecology, University of GöttingenGöttingen, Germany; ^2^Faculty of Agriculture, Jenderal Soedirman UniversityPurwokerto, Indonesia; ^3^Bioclimatology, University of GöttingenGöttingen, Germany; ^4^Faculty of Forestry, Bogor Agricultural UniversityBogor, Indonesia

**Keywords:** heterogeneity, Indonesia, sap flux, Sumatra, transpiration, variability

## Abstract

Oil palm and rubber plantations extend over large areas and encompass heterogeneous site conditions. In periods of high rainfall, plants in valleys and at riparian sites are more prone to flooding than plants at elevated topographic positions. We asked to what extent topographic position and flooding affect oil palm and rubber tree water use patterns and thereby influence spatial and temporal heterogeneity of transpiration. In an undulating terrain in the lowlands of Jambi, Indonesia, plantations of the two species were studied in plot pairs consisting of upland and adjacent valley plots. All upland plots were non-flooded, whereas the corresponding valley plots included non-flooded, long-term flooded, and short-term flooded conditions. Within each plot pair, sap flux densities in palms or trees were monitored simultaneously with thermal dissipation probes. In plot pairs with non-flooded valleys, sap flux densities of oil palms were only slightly different between the topographic positions, whereas sap flux densities of rubber trees were higher in the valley than at the according upland site. In pairs with long-term flooded valleys, sap flux densities in valleys were lower than at upland plots for both species, but the reduction was far less pronounced in oil palms than in rubber trees (-22 and -45% in maximum sap flux density, respectively). At these long-term flooded valley plots palm and tree water use also responded less sensitively to fluctuations in micrometeorological variables than at upland plots. In short-term flooded valley plots, sap flux densities of oil palm were hardly affected by flooding, but sap flux densities of rubber trees were reduced considerably. Topographic position and flooding thus affected water use patterns in both oil palms and rubber trees, but the changes in rubber trees were much more pronounced: compared to non-flooded upland sites, the different flooding conditions at valley sites amplified the observed heterogeneity of plot mean water use by a factor of 2.4 in oil palm and by a factor of 4.2 in rubber plantations. Such strong differences between species as well as the pronounced heterogeneity of water use across space and time may be of relevance for eco-hydrological assessments of tropical plantation landscapes.

## Introduction

Oil palm (*Elaeis guineensis* Jacq.) and rubber (*Hevea brasiliensis* Müll. Arg.) plantations cover large areas in tropical regions (FAO, 2016) and are projected to expand further (Fox et al., 2012; Van der Laan et al., 2016). In contrast, the area covered by natural forests has strongly declined over the last decades (Keenan et al., 2015). From environmental perspectives, this raises concerns not only with respect to biodiversity (Barnes et al., 2014) but also regarding the integrity of the hydrological cycle including potential changes in transpiration (Ziegler et al., 2009; Sterling et al., 2012).

In previous studies, oil palm transpiration rates were analyzed on 15 on-farm plots in maritime Indonesia using a sap flux technique in conjunction with eddy covariance measurements at two sites (Röll et al., 2015; Meijide et al., 2017). Oil palm water use and transpiration increased from young to about 8-year-old plantations and then leveled off up to an age of 22 years (Röll et al., 2015). Among medium-aged, 10–18 years old plantations substantial spatial heterogeneity was found. The highest oil palm stand (evapo) transpiration rates were observed in an intensively managed plantation; they were as high as those of rainforests in the same region (Röll et al., 2015; Meijide et al., 2017). It was also indicated that the temporal dynamics of transpiration in oil palm are “buffered,” which means that day-to-day transpiration rates fluctuate less than micrometeorological drivers (Röll et al., 2015). For rubber plantations, previous studies reported relatively high evapotranspiration rates (i.e., partly higher than in adjacent natural forests) from China, Thailand, and Cambodia by means of eddy covariance measurements (Tan et al., 2011; Giambelluca et al., 2016). At the site in Cambodia, an experimental farm, both sap flux (Kobayashi et al., 2014) and eddy covariance measurements (Giambelluca et al., 2016) indicated that rubber tree transpiration responds sensitively to dynamics in radiation, air humidity and soil moisture.

In South East Asia and particularly Indonesia, oil palm and rubber plantations extend over large areas and thus commonly encompass heterogeneous sites. Vast parts of the Southeast Asian lowlands are actually not flat but undulating, separating into upland and valley sites (Miettinen et al., 2014). This leads to differences in soil moisture regimes: when rainfall is low, soil moisture availability for plants will be higher at valley sites (Sauer et al., 2005; Tromp-van Meerveld and McDonnell, 2006). Under prevailing high rainfall, plants in valleys and at riparian sites are often flooded for different durations, whereas plants at higher elevations on uplands sites are less prone to flooding (Deo et al., 2015).

In North-America, upland-to-wetland gradients were analyzed by means of sap flux measurements in order to evaluate the significance of site conditions for tree and stand transpiration (Loranty et al., 2008; Mackay et al., 2010; Angstmann et al., 2012). Pronounced differences in tree transpiration were observed among sites and it was concluded that it is necessary to include plots at different topographic positions for landscape-level analyses or modeling of transpiration. Sap flux density and stand transpiration of Japanese cypress (*Chamaecyparis obtusa*) were significantly higher at valley than at upland sites (Kume et al., 2015), while being similar at both locations for Japanese cedar (*Cryptomeria japonica*) (Kumagai et al., 2008). For tropical rainforest regions such studies are still rare, but the influence of the water table on certain species was analyzed in northern Australia (McJannet, 2008) and Hawaii (Santiago et al., 2000). It was reported that waterlogging reduces transpiration of some species, while other species can adapt to this condition and were thus not or less affected. In tropical rainforests, tree species composition often differs between upland and periodically wet or riparian sites. Species on periodically wet or riparian sites (e.g., *Melaleuca argentea* W. Fitzg. and *Corymbia bella* Hill) may have the ability to transpire at high rates even during inundation due to adaptations, e.g.,. in the root system (O’Grady et al., 2006). In contrast, there is no difference in species composition across sites and topographic positions in monoculture plantations such as rubber and oil palm.

To our knowledge, whether, and if so to what extent landscape position and flooding induce spatial heterogeneity and temporal variability in transpiration rates of oil palms and rubber trees is so far unknown. Our study was implemented in the lowlands of Jambi province on Sumatra, Indonesia. The region is characterized by a steadily undulating topography (Gouyon et al., 1993; Whitten et al., 2000). Only a few decades ago, the region was largely covered by rainforests; today, it is dominated by oil palm and rubber plantations (Laumonier et al., 2010; Margono et al., 2012). Our objective was to determine to what extent topographic position and flooding affect oil palm and rubber tree water use patterns and thereby influence spatial and temporal heterogeneity of transpiration. The study may thus contribute to an improved eco-hydrological assessment of post-forest plantations landscapes in tropical lowlands.

## Materials and Methods

### Study Region

**FIGURE 1:**
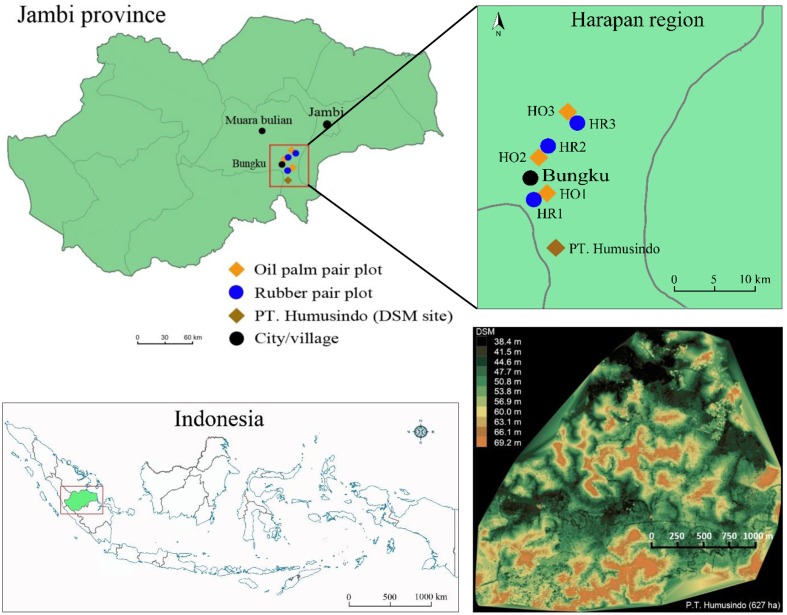
**The study region (“Harapan region”) in Jambi Province, Sumatra, Indonesia**. Location of oil palm and rubber plot pairs (each consisting of upland and valley sites). Upland sites were part of the larger experimental design of the EFForTS project (Drescher et al., 2016) and had plot codes H (for Harapan) with O for oil palm and R for rubber; numbering goes from South to North. The terrain of the landscape is undulating, as to be seen on a digital surface model (DSM, Naumann, 2015) of a 627 ha region in an oil palm plantation owned by P. T. Humusindo, approximately 10 km south of our study region (indicated by brown rhombus).

The study was carried out close to the equator in the “Harapan region” of the lowlands of Jambi Province, Sumatra, Indonesia (**Figure 1**). Annual precipitation averages 2235 mm and average temperature is 26.7°C (Drescher et al., 2016). The terrain is undulating with altitudes varying between 40 and 100 masl at relatively short distance (**Figure 1**).

### Study Sites

**FIGURE 2:**
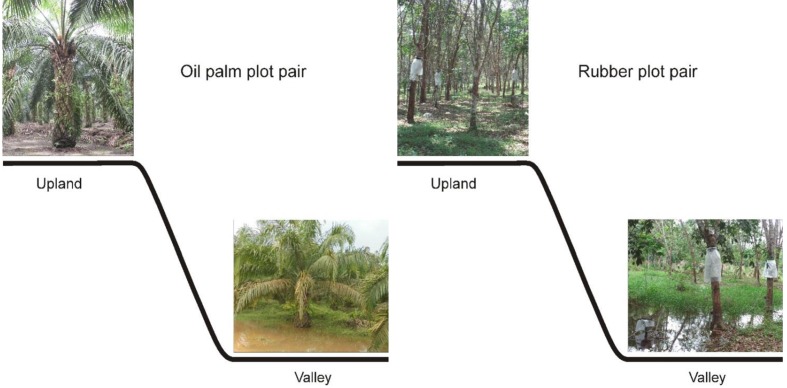
**In oil palm and rubber plantations, plot pairs consisting of upland and adjacent valley sites were studied, with three replicates for each plantation type**. In the study period, the upland plots were never flooded, whereas the valley plots comprised non-flooded, long-term flooded, and short-term flooded conditions. Within each plot pair, sap flux measurements were conducted simultaneously at the upland and according valley site.

Our study comprised 12 plots in six plot pairs. For each plantation type, oil palm or rubber, we studied three plot pairs. Within each pair, one plot was situated at an upland site and the other at an adjacent valley site (**Figure 2**). The upland and valley sites were not more than 50 m apart. The upland plots were part of the general experimental design of the EFForTS project (Drescher et al., 2016; nomenclature: HO1, HO2, HO3 and HR1, HR2, HR3, where H stands for Harapan region, O for oil palm and R for rubber); the soil type at upland plots is loam Acrisol (Drescher et al., 2016). Valley sites have alluvial soils formed by accumulating eroded uphill soils. During the entire study period the upland plots were never flooded. During 4-week measurement periods, the valley plots ranged from non-flooded over short-term flooded (4–5 days) to long-term flooded (>22 days, **Table 1**). The plantations were between 14 and 18 years old. Stand densities tended to be higher at the upland sites, whereas diameters were similar between the corresponding upland and valley plots (**Table 1**). The plantations were owned and managed by local smallholders who, within a given plantation type, applied similar cultivation practices across sites.

**Table 1:** Stand characteristics and moisture conditions at the different topographic positions in oil palm and rubber plantations.

	Oil palm plantation		Rubber plantation	
	Upland	Valley	Upland	Valley
Age (years)	16 ± 1.5	16 ± 1.5	15 ± 1.5	15 ± 1.5
Density (trees ha^-1^	144 ± 6.9	132 ± 2.6	380 ± 55.4	354 ± 103.6
Diameter (cm)	80.1 ± 4.0	77.6 ± 3.0	22.8 ± 3.8	24.5 ± 3.1
Sap wood area (cm^-2^)^a^	25.3 ± 1.7	22.1 ± 0.9	181.6 ± 10.0	219.5 ± 6.1
Numbers of leaves	34 ± 2	38 ± 3	–	–
Height (m)	5.7 ± 1.6	5.6 ± 0.9	14.4 ± 0.2	15.0 ± 0.5
Soil moisture (%), non-flooded^b^	26.2 ± 4.4	38.1 ± 1.2	32.8 ± 2.3	37.8 ± 3.7
Flooded (days)	HO2 0	0	HR2 0	0
	HO1 0	4	HR3 0	5
	HO3 0	30	HR1 0	22

*Means and standard errors, *n* = 3.*

^a^*Sap wood area of oil palm (average per leaf) and rubber trees (average per stem)*.

^b^*n = 2 for valley sites, mean of soil moisture at non-flooded oil palm (HO2) and rubber (HR2) valley sites and soil moisture at short-term flooded valley sites (HO1 and HR3) under non-flooded conditions. Short- and long-term flooded conditions are excluded*.

### Sap Flux Measurements and Transpiration

For each plot pair, sap flux densities (*J*_s_, g cm^-2^ h^-1^) were measured simultaneously at upland and valley plots with Granier-type thermal dissipation probes (TDP, Granier, 1985) for at least 4 weeks. For oil palm, we followed the sampling and data processing procedure as described in Niu et al. (2015). Four oil palms, each with four sensors inserted into individual leaf petioles, were equipped with TDPs. Specific calibration parameters were applied to calculate *J*_s_ (Niu et al., 2015) and leaf conductive areas (cm^2^) were determined from a linear regression with petiole baseline length (Niu et al., 2015). To calculate palm water use rates (WU, kg day^-1^), respective sap flux densities were multiplied by average leaf conductive areas and by the average number of leaves per palm. Multiplying the resulting average palm water use by the number of palms per unit of land yielded stand transpiration rates (*T*; mm day^-1^). All 16 TDP sensors of a certain oil palm plot running simultaneously would result in an error margin of estimated stand transpiration of oil palms of about 10% (Niu et al., 2015). This error is due to a limited sample size for both, establishing the average water conductive area per palm (by counting leaves and measuring petiole baseline length on a limited number of palms) and establishing average *J*_s_ (with a limited number of TDP sensors running simultaneously). Stand transpiration as the product of stand water conductive area and *J*_s_ reflects both of these errors.

For rubber trees, TDP sensors were installed on six trees per plot with two sensors per tree. The sensors were inserted above the latex harvesting area at a height of about 2–2.5 m. *J*_s_ was calculated using the original Granier (1985) equation, which was confirmed in a calibration experiment (Niu et al., unpublished). Recently established radial *J*_s_ profiles accounting for changes in *J*_s_ patterns with increasing xylem depth in rubber trees were applied (Niu et al., unpublished) to calculate tree water use (kg day^-1^). Using inventory data from the EFForTS project (stand density and tree diameters, Kotowska et al., 2015), stand transpiration rates could be calculated for the upland sites (see Niu et al., unpublished, for details); at adjacent valley sites, tree diameter and tree distance were recorded with a measurement tape. According to Kume et al. (2010) potential estimation errors in stand transpiration rates stem from both, a limited sample size (i.e., number of sensors) for establishing the average water conductive area and for establishing average *J*_s_. For our rubber field measurement scheme, i.e., 12 sensors running simultaneously on six trees error margins of average *J*_s_ have been reported to be about 10% (Kobayashi et al., 2014).

### Soil Water Content

Soil water content was measured with time domain reflectometry (TDR) sensors (CS616, Campbell Scientific, UK). We installed eight TDRs per plot at two trees or palms, at 1 and 2 m distance from the trunk, and at two depths (0–30 and 30–60 cm). Data were recorded hourly by a data logger (CR1000, Campbell Scientific, UK). Flooding was observed visually.

### Micrometeorological Variables

Micrometeorological variables were monitored at a station about 10 km distant from our study plots in open terrain. Air temperature and humidity were measured by thermohygrometers (type 1.1025.55.000, Thies Clima, Germany) and were used to calculate vapor pressure deficit (VPD, kPa). Global radiation (*R*_g_, MJ m^-2^ h^-1^) was measured using a CMP3 pyranometer (Kipp & Zonen, Delf, Netherlands). Measurements were taken every 15 s and averaged and stored as 10 min values on a data logger (type DL16 Pro, Thies Clima, Germany).

### Data Analysis

Even though 4-week data series of transpiration were available for each plot pair, results are presented mainly for selected sunny days (radiation >17 MJ m^-2^ day^-1^; VPD daytime average >1.1 kPa) only. In the case of the short-term flooded plots, naturally only a few days of data were available under flooded conditions; to make these comparable to non-flooded or long-term flooded valley sites, we thus chose to focus on a sunny day only, in order to reduce influences of varying weather conditions among the different measurement periods. For plot pairs with non-flooded or long-term flooded valley sites, for which longer data series were available, the mean values of three sunny days were used to increase the robustness of the results. As most plot pairs were measured successively rather than simultaneously and thus potentially encompassed greatly varying weather conditions, using sunny days helped to focus the analysis on the spatial heterogeneity of (stand) transpiration as induced by flooding, rather than on the temporal day-to-day dynamics of transpiration.

Results are presented both as daily values and as diurnal hourly means; as a measure of the spatial, within-plot variability of *J*_s_ and stand transpiration, standard errors (SE) of these values are provided; they were derived among all trees or palms within a certain plot (i.e., from 16 sensors on four oil palms or 12 sensors on six rubber trees, in analogy to Niu et al., 2015). Maximum *J*_s_ values (*J*_smax_) were derived from the 90-percentile of hourly *J*_s_ observations of the selected sunny days. Because of unequal variances, we used Welch’s *t*-test to test for significant differences (*P* < 0.05) in *J*_smax_ and average tree/palm water use within plot pairs, i.e., between upland and valley sites. Likewise, we used Welch’s *t*-test (applicable for time repeated measurements) to test for differences between flooded and non-flooded conditions at the short-term flooded valley sites.

The day-to-day *J*_s_ responses to VPD and *R*_g_ were analyzed with a power function. Statistical analyses were performed with R version 3.0.2.

## Results

**FIGURE 3:**
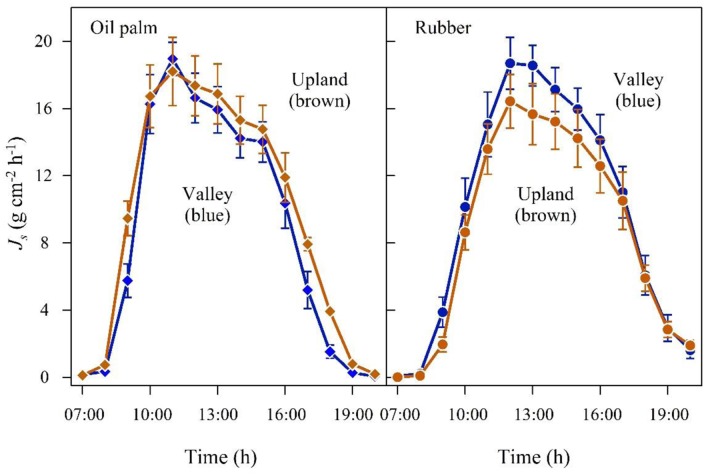
**Oil palm **(left)** and rubber **(right)** plot pairs with non-flooded valley**. Diurnal course of sap flux density (*J*_s_) at the upland (brown) and corresponding non-flooded valley site (blue). Hourly means of three sunny days; vertical bars show the standard error of the mean in sap flux among rubber trees (*n* = 6 trees; each with two sensors) and oil palms (*n* = 4 palms; each with four leaves and one sensor per leaf) in a plot at a given time.

In the plot pairs with non-flooded valleys, oil palm *J*_smax_ and daily accumulated *J*_s_ were 4 and 12% higher in the valley than on the upland plot (**Figure 3** and **Table 2**). The differences were more pronounced in the rubber plot pair; *J*_smax_ and the daily accumulated *J*_s_ were 16 and 30% higher in the valley (**Figure 3** and **Table 2**). These differences go along with higher soil moisture contents at valley plots (oil palm: 28 vs. 37%; rubber: 30 vs. 40%, **Table 1**).

**Table 2:** Maximum sap flux density (*J*_smax_) in oil palm leaf petioles and rubber tree trunks, water use per palm and tree and estimated stand transpiration rates at upland and valley plots under varying flooding conditions, i.e., non-flooded, long-term flooded, and short-term flooded (sunny days, means and standard errors).

Plot pair	Oil palm			Rubber		
	*J*_smax_ (g cm^-2^ h^-1^)	Water use (kg day^-1^)	Transpiration (mm day^-1^)	*J*_smax_ (g cm^-2^ h^-1^)	Water use (kg day^-1^)	Transpiration (mm day^-1^)
Valley non-flooded						
Upland (HO2 and HR2)	17.1 ± 0.9	94.8 ± 4.5	1.3	16.7 ± 0.7	26.6 ± 3.2	0.9
Valley	17.7 ± 0.9ˆns1	94.7 ± 4.6ˆns1	1.2	19.3 ± 0.7ˆns1	33.2 ± 2.5*1	1.2
Valley long-term flooded						
Upland (HO3 and HR1)	14.8 ± 0.5	97.0 ± 5.0	1.4	15.3 ± 1.0	26.5 ± 3.2	0.9
Valley	11.6 ± 0.6*1	63.0 ± 2.0*1	0.8	8.4 ± 0.5**1	13.8 ± 2.1**1	0.6
Valley short-term flooded						
Upland (HO1 and HR3)	13.6 ± 0.5	83.9 ± 2.5	1.2	17.8 ± 0.6	21.8 ± 1.9	1.0
Valley						
1. Non-flooded	16.1 ± 0.6ˆns2	81.3 ± 3.9ˆns2	1.1	21.3 ± 0.9*2	34.0 ± 2.6*2	0.9
2. Flooded	16.0 ± 0.6	82.7 ± 3.5	1.1	17.3 ± 0.7	27.8 ± 2.1	0.7

*Asterisks indicate significant differences between upland and valley sites^1^ and between flooded and non-flooded condition at the short-term flooded valley sites^2^ at ^∗^*P* < 0.05 and ^∗∗^*P* < 0.01; ns, not significant, time repeated *t*-tests*.

**FIGURE 4:**
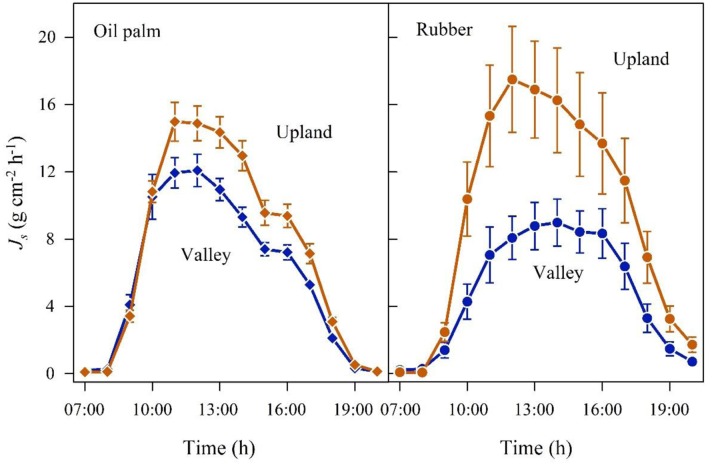
**Oil palm **(left)** and rubber **(right)** plot pairs with long-term flooded valley**. Diurnal course of sap flux density (*J*_s_) at the upland (brown) and corresponding long-term flooded valley site (blue). Hourly means of three sunny days; vertical bars show the standard error of the mean in sap flux among rubber trees (*n* = 6 trees; each with two sensors) and oil palms (*n* = 4 palms; each with four leaves and one sensor per leaf) in a plot at a given time.

**FIGURE 5:**
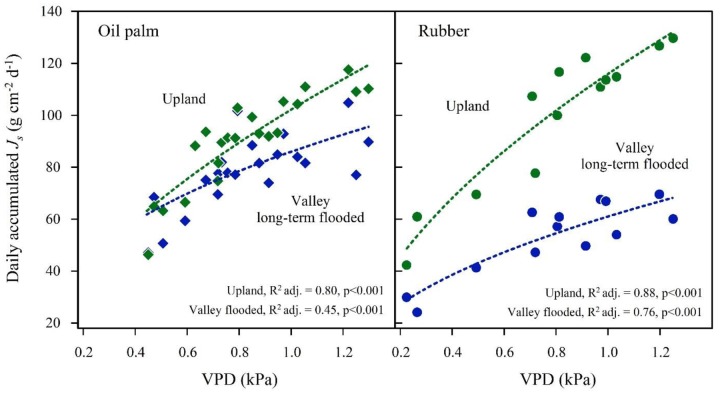
**Oil palm **(left)** and rubber **(right)** plot pairs with long-term flooded valley**. Daily accumulated sap flux density (*J*_s_) in response to changes in average daily vapor pressure deficit (VPD) at the upland (green) and corresponding long-term flooded valley site (blue). Plot pairs were HO3 for oil palm and HR1 for rubber.

**FIGURE 6:**
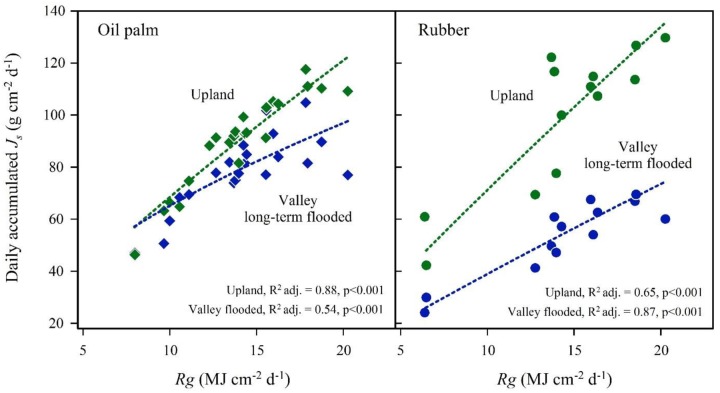
**Oil palm **(left)** and rubber **(right)** plot pairs with long-term flooded valley**. Daily accumulated sap flux density (*J*_s_) in response to changes in global radiation day sums (*R*_g_) at the upland (green) and corresponding long-term flooded valley site (blue). Plot pairs were HO3 for oil palm and HR1 for rubber.

In the plot pairs with long-term flooded valleys, oil palm in the valley showed lower *J*_s_ than at upland plots (**Figure 4**). The *J*_smax_ in the valley was by 22% lower than on the upland plot (**Table 2**). A similar pattern was found in rubber trees, but again the difference was more pronounced (*J*_smax_ -45%). Long-term flooding also influenced the day-to-day *J*_s_ response to changes in VPD and *R*_g_: accumulated daily *J*_s_ generally increased with increasing VPD (**Figure 5**) and *R*_g_ (**Figure 6**) for both plantation types and flooding conditions, but the slopes of the regression equations were smaller in valleys than at upland sites, particularly for rubber (**Figures 5**, **6**).

**FIGURE 7:**
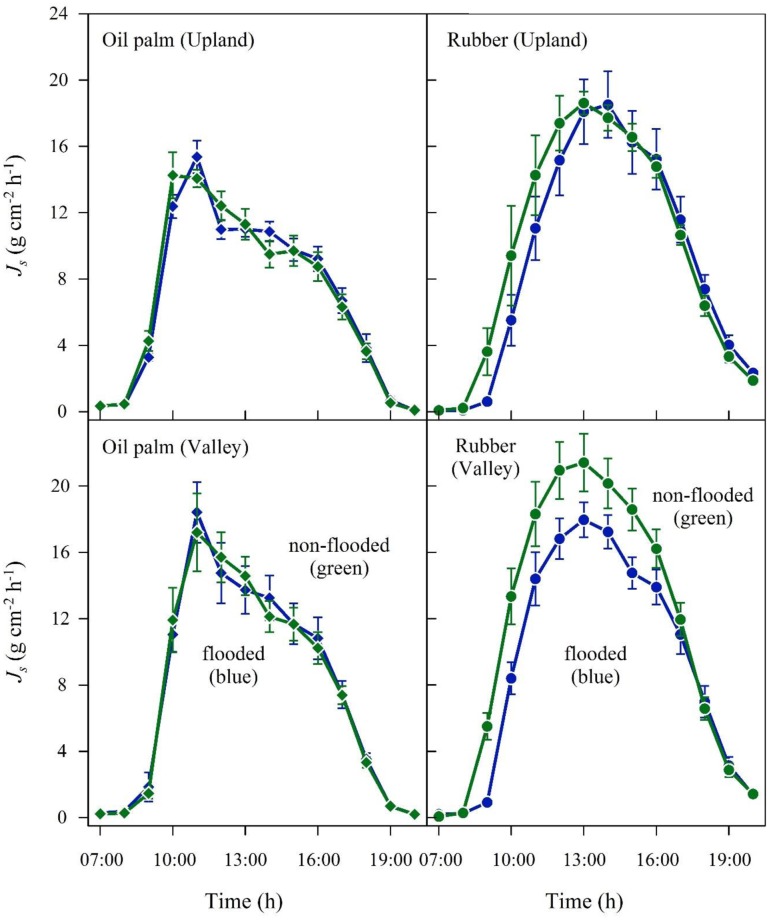
**Oil palm **(left)** and rubber **(right)** plot pairs with short-term flooded valley**. Flooded and non-flooded conditions at the valley sites are compared. Diurnal course of sap flux density (*J*_s_) at upland **(upper)** and corresponding valley sites **(lower)**. Upland sites were always non-flooded, but valley sites varied between non-flooded (green) and short-term flooded (blue) conditions. For the according periods (i.e., valley non-flooded, valley short-time flooded), upland *J*_s_ as a reference is displayed separately (in green and blue, respectively). Hourly values on a sunny day; vertical bars show the standard error of the mean in sap flux among rubber trees (*n* = 6 trees; each with two sensors) and oil palms (*n* = 4 palms; each with four leaves and one sensor per leaf) in a plot at a given time.

In the plot pair with short-term flooded valley, *J*_s_ in oil palm hardly responded to flooding (**Figure 7** and **Table 2**). In contrast, *J*_smax_ and daily accumulated *J*_s_ in rubber were 19 and 22% lower on a flooded day than on a non-flooded day, indicating a much more sensitive response to short-term flooding in rubber trees than in oil palms.

**FIGURE 8:**
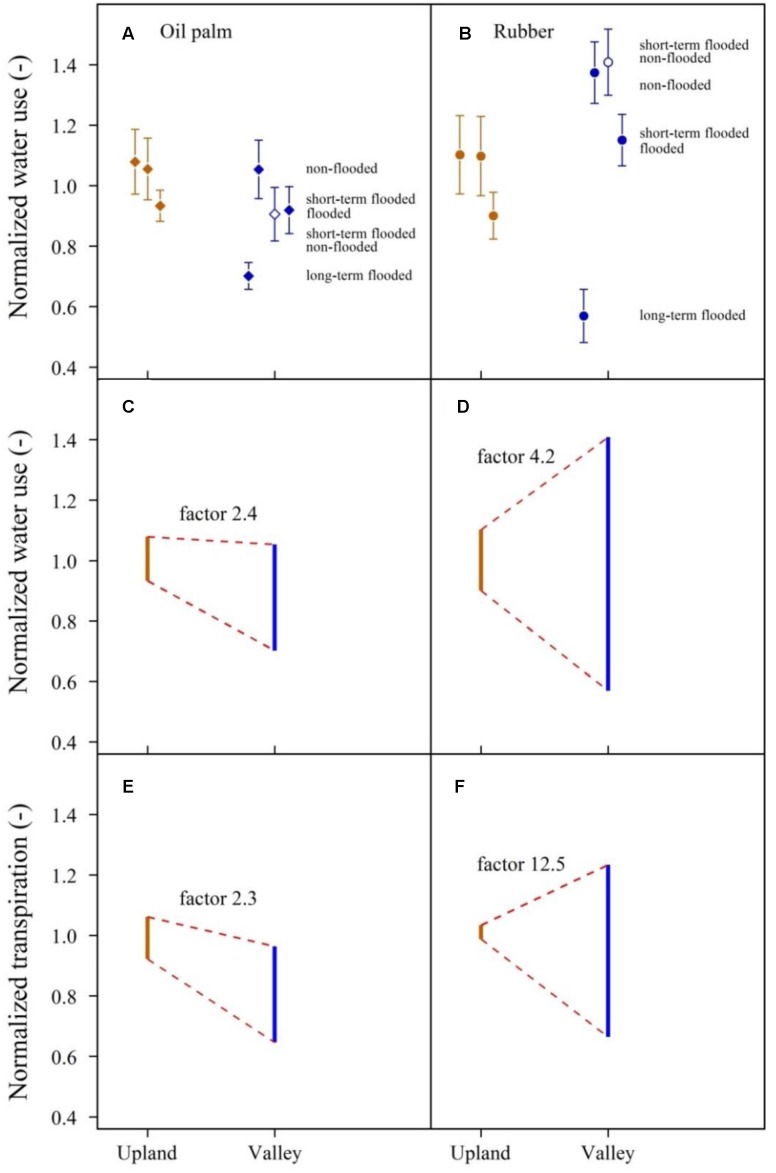
**Long-term, short-term, and non-flooded plot pairs combined**. Water use by oil palm and rubber trees across upland and valley plots, normalized by mean oil palm **(A)** and rubber tree **(B)** water use on the upland plots (sunny days, means and standard errors). Short-term flooded valley plots are given twice, once under flooded (closed symbols) and once under non-flooded (open symbols) conditions. Dynamics of the heterogeneity of normalized water use **(C,D)** and normalized transpiration **(E,F)** of oil palm and rubber from upland to valley plots. Displayed factors quantify the heterogeneity that is induced as a result of topographic position and flooded conditions at valley sites in relation to the heterogeneity at upland sites.

Overall, topographic position and flooding affected the heterogeneity of rubber tree and oil palm water use differently. The observed range of plot-averaged, normalized oil palm water use was 2.4-fold larger at valley sites than at upland sites, while for rubber trees, it was 4.2-fold larger at valley sites (**Figure 8** and **Table 2**). Translating these differences from the tree/palm level to patterns of transpiration estimates at the plot scale, i.e., taking into account differences in tree and palm densities and sizes between upland and valley sites, the heterogeneity of transpiration was increased by a factor of 2.3 from the upland to the valley in oil palm and by a factor of 12.5 in rubber plots (**Figure 8** and **Table 2**).

## Discussion

In our study, topography and flooding induced substantial heterogeneity in plant water use. This was more pronounced in rubber trees than in oil palms. Our estimates of stand transpiration (0.6–1.2 mm day^-1^ for rubber, 0.8–1.4 mm day^-1^ for oil palm) were substantially lower than those reported for rubber plantations on the Asian mainland (∼2 mm day^-1^, Isarangkool Na Ayutthaya et al., 2010; Kobayashi et al., 2014; Sopharat et al., 2015) and lower than those reported for an intensively managed oil palm plantation in the study region (2.5–2.7 mm day^-1^; Röll et al., 2015; Meijide et al., 2017). However, our oil palm estimate falls into the range of 1.1–2.5 mm day^-1^ from a previous study in 12 productive oil palm plantations in the study region (including our three upland oil palm sites, Röll et al., 2015). Likewise, our rubber estimate compares to the mean of five-fully leaved, mature plantations in the study region (1.3 mm day^-1^, Niu et al., unpublished). Potential reasons for partly relatively large differences between our values and those reported in other studies include differences among methods with respect to the cited rubber studies, as well as different environmental site conditions and differences in management practices such as the utilization of varying clone types. In oil palm, our measurement approach has been calibrated (Niu et al., 2015) and yielded plausible results when compared to eddy covariance data (Röll et al., 2015). For rubber, no eddy covariance data was available for comparison, but conducted laboratory experiments and field assessments with different sap flux techniques showed no contradictions and give confidence that our approach is feasible (Niu et al., unpublished). Analyses of estimation uncertainties of water use rates due to limited sample size with our respective oil palm and rubber field measurement schemes point to potential uncertainties of mean water use of about 10% (Kobayashi et al., 2014; Niu et al., 2015). However, and regardless of potential uncertainties in absolute water use rates of rubber trees and oil palms, this study was mainly designed to investigate relative differences in water use patterns between topographic positions and flooding conditions; to accurately quantify such relative differences in patterns is regarded a strength of sap flux approaches.

For both oil palms and rubber trees, *J*_s_ and plant water use were higher at non-flooded valley sites than at the corresponding upland sites. This was probably caused by more favorable soil moisture contents in valleys (i.e., higher, but not waterlogged, **Table 1**), which are likely due to run-off and drainage from adjacent slopes. In contrast, *J*_s_ and water use of both plantation types were lower than at upland plots in long-term flooded valleys. Floods reduce gaseous oxygen concentrations in soils and thus lead to hypoxic or anoxic conditions (Ezin et al., 2010; Wittmann and Pfanz, 2014). Root hydraulic conductivity and permeability are severely reduced (Else et al., 2001; Parent et al., 2008; Kreuzwieser and Rennenberg, 2014), which results in changes in leaf water potential, stomatal closure, and lower leaf-specific hydraulic conductivity (Else et al., 2001; Atkinson et al., 2008; Herrera, 2013; Zhao et al., 2014). Consequently, plant water uptake is often substantially reduced under flooded conditions (Nicolás et al., 2005; Aroca et al., 2012; Yan et al., 2015). Long-term flooding can furthermore decrease water uptake capacities by injuring roots (Drew, 1997). For a scenario of frequent, long-term flooding, e.g., at riparian or valley sites, damages to the trunk induced by the frequent manual cutting for latex extraction could be enhanced, particularly at the trunk base. This could facilitate the entry of fungi and potentially reduce productivity and vitality of flood-prone rubber trees. However, flood-related mortality of rubber trees was not observed in the study region (personal observation); this is likely due to a combination of the relatively short rotation cycle of monoculture rubber plantations in the study region (approximately 20 years) and adaptions to regular flooding in the natural habitat of rubber (Amazonia). In accordance with the substantial decreases in the water use of oil palms (-35%) and rubber trees (-48%) under long-term flooded conditions in our study, decreases in water uptake under long-term flooded conditions were also pronounced in studies on other (tropical) species such as apricot (∼15%), eucalyptus (∼20%), and lemon (∼50%) trees (Nicolás et al., 2005; Ortuño et al., 2007; Miyazawa et al., 2014).

Theoretically, lower (stand) transpiration of both plantation types under long-term flooded conditions could also be due to factors other than flooding, e.g., differences in site or stand characteristics. As the flooding periods at the long-term flooded valley sites exceeded the respective sap flux measurement periods, no data are available to evaluate stand transpiration under non-flooded conditions. However, the results from the short-term flooded plots for both oil palm and rubber (**Figure 7**) do not contradict the finding of lower transpiration as a result of long-term flooding. Further, the respective upland and valley sites of each plot pair (and thus including the pairs with long-term flooded valley) were located close to each other (<50 m distance), within the same (small-holder) plantations. Non-flooding related additional factors such as site, stand or management characteristics within plot pairs were thus relatively homogeneous and unlikely to be the dominant factor for low stand transpiration rates at long-term flooded valleys sites. Asides from the substantially reduced water use rates, the strong influence of long-term flooding on plant water use in our study is also visible in the less sensitive responses of *J*_s_ to VPD and radiation (**Figures 5**, **6**). These micrometeorological drivers were not measured above the canopy of each particular site but rather represent more general, open area upland conditions some kilometers from our sites. However, while variables such as air humidity and wind speed likely vary considerably between upland and valley sites even at short distance, radiation as an important driver of transpiration can be expected to vary far less due to the close proximity of our study sites to the equator. Keeping this in mind, our results indicate a more conservative response to environmental drivers at long-term flooded valley sites for both species, which may be related to reductions in root hydraulic conductivity and stomatal conductance. Substantial decreases in stomatal conductance were, e.g., reported for sweet gum (*Liquidambar styraciflua* L.), where it decreased by 24% after 1 day of flooding and by 70% after 9 days of flooding (Pezeshki and Chambers, 1985).

For all of the topographic positions and flooding conditions evaluated in this study, *J*_s_ and water use of rubber trees responded more sensitively than *J*_s_ and water use of oil palms. Varying *J*_s_ responses to flooded conditions between oil palm and rubber are in line with results from other studies, e.g., strong *J*_s_ decreases in *Eucalyptus camaldulensis*, but no noticeable reductions of *J*_s_ in the *Shorea roxburghii* and *Dipterocarpus obtusifolius* (Miyazawa et al., 2014). Likewise, *J*_s_ was reported to be higher at valley sites than at upland sites for Japanese cypress (Kumagai et al., 2008), while being similar at both locations for Japanese cedar (Kume et al., 2015).

Such differences between species in their water use response to flooding are influenced by differences in plant morphology, particularly of the root and leaf system (Aroca et al., 2012; Kreuzwieser and Rennenberg, 2014; Miyazawa et al., 2014). Oil palm has a fibrous root system and its roots can spread over 6 m vertically and 25 m horizontally, while the rubber root system typically extends less (3–13 m) (Jourdan et al., 2000; Gonkhamdee, 2010). On an intra-annual basis, oil palms are not very dynamic in leaf area. The water use response of oil palms at upland sites to environmental drivers, in particular to air humidity, has been described as buffered (Röll et al., 2015). Based on hysteresis between water use and VPD, it was argued that internal stem water storage herein plays a role, as it had previously been observed in other palm species (Holbrook and Sinclair, 1992). In contrast to these attributes of oil palm, rubber trees at upland sites are quite responsive to dynamics of environmental drivers and shed their leaves during dry seasons (Kobayashi et al., 2014; Giambelluca et al., 2016), both of which enhances the heterogeneity of rubber water use across space and time. Within certain ranges, our findings thus point to and strengthen previous suggestions that oil palm water use is only moderately affected by environmental conditions including floods. In contrast, rubber tree water use is quite responsive to fluctuations in environmental conditions including short- and long-term flooding.

Both rubber and oil palm plantations cover large areas of the Sumatran lowlands, where they have replaced highly diverse natural forests. It, however, seems that the post-forest plantation landscape has more heterogeneous transpiration patterns than one might expect. This heterogeneity can partly be explained by the age class structure of the plantation landscape and by species differences in the response to environmental drivers including periodical leaf shedding in rubber (Röll et al., 2015; Niu et al., unpublished). As to how far different management schemes also play a role needs to be analyzed in more depth. Patterns described in Röll et al. (2015) suggest that high fertilizer input as mostly found in large estates leads to higher transpiration rates than in less intensively managed smallholder plantations. The present study adds yet another dimension to the plantation landscape by suggesting that topography and flooding are also strong factors influencing the heterogeneity of landscape-level transpiration patterns. Likewise, studies investigating upland-to-wetland gradients in North-America also found pronounced differences in tree transpiration and it was concluded that it is necessary to include sites at different topographic positions for landscape-level analyses or modeling (Loranty et al., 2008; Mackay et al., 2010; Angstmann et al., 2012). In our case, we found only moderate variation in oil palm water use across space and time, whereas rubber responded strongly to topographic position and temporal flooding. These differences may be of interest in eco-hydrological assessments of post-forest plantation landscapes.

## Author Contributions

The concept and research priorities for this study on rubber trees in Sumatra were developed by DH, in close cooperation with H. Setup and maintenance of the field installations and data collection were performed by AH, AR, FN, and AM. Data analysis, plotting, and manuscript writing were mainly carried out by AH, AR, FN, and DH, in close cooperation with the other co-authors.

## Conflict of Interest Statement

The authors declare that the research was conducted in the absence of any commercial or financial relationships that could be construed as a potential conflict of interest.
